# A Doctor Well Preserved

**Published:** 1869-04

**Authors:** 


A DOCTOR WELL-PRESERVED.—We had the pleasure of meeting Dr. Wayland, Sen., of Clark Co., Mo., a few days ago, in consultation. We have known the Doctor by reputation for many years, but have never met him professionally until now. The Doctor is in his eighty-third year, has been engaged in the practice of his profession some fifty-two years. He removed from Virginia to his present location in Missouri, thirty-three years ago, and has been actively engaged in his profession ever since. He is well-preserved, both mentally and physically, and from present appearances might live a score of years longer.

